# Mobile phone brief intervention applications for risky alcohol use among university students: a randomized controlled study

**DOI:** 10.1186/1940-0640-8-S1-A8

**Published:** 2013-09-04

**Authors:** Anne H Berman, Mikael Gajecki, Kristina Sinadinovic, Morgan Fredriksson, Charlie Lindviken, Claes Andersson

**Affiliations:** 1 Karolinska Institute, Department of Clinical Neuroscience, Center for Psychiatric Research, Stockholm, Sweden; 2 KTH Royal Institute of Technology, Department of Speech, Music and Hearing, Stockholm, Sweden; 3 Stockholm Center for Dependency Disorders, Stockholm, Sweden; 4 Malmö University, Malmö, Sweden

## 

A large proportion of university students overconsume alcohol. Brief interventions via internet and automated telephony have been shown to reduce students’ alcohol intake. This study tested the efficacy of two mobile phone applications (apps) targeting individual drinking choices on party occasions, with the goal of reducing problematic alcohol intake among urban Swedish university students. Students were recruited via e-mails to all student union members at two large universities. Those who gave informed consent, had a smart phone, and showed risky alcohol consumption according to the Alcohol Use Disorders Identification Test (AUDIT) were included and randomized into 3 groups. Group 1 had access to a Swedish state monopoly of alcoholic beverages app offering real-time blood alcohol count (BAC) calculation; Group 2 had access to a web-based app developed by the research group, offering real-time BAC calculation with the addition of planning and follow-up functions covering alcohol intake; Group 3 participants were controls. All groups were followed up after 7 weeks, including participants without risky alcohol use. Among 28591 students offered participation, 4823 agreed to join the study; 414 were excluded due to incomplete baseline data, and 1932 fulfilled eligibility criteria for randomization, with 643, 640 and 649 participants in each of groups 1, 2 and 3. After 7 weeks, attrition was 28% in Group 1, 41% in Group 2, 25% in Group 3, and 21% among non-risky use participants. Outcome analyses for alcohol are reported, taking into account the regression to the mean phenomenon. In addition, qualitative usability data are presented. Mobile phone apps appear offer a huge potential value for making state-of-the-art brief interventions available to more university students than ever before. The results of this study will be useful for future research with university students as well as problematic alcohol users in other contexts.

